# Comparison of CT, MRI, and F-18 FDG PET/CT for initial N-staging of oral squamous cell carcinoma: a cost-effectiveness analysis

**DOI:** 10.1007/s00259-022-05843-4

**Published:** 2022-05-24

**Authors:** Egon Burian, Benjamin Palla, Nicholas Callahan, Thomas Pyka, Constantin Wolff, Claudio E. von Schacky, Annabelle Schmid, Matthias F. Froelich, Johannes Rübenthaler, Marcus R. Makowski, Felix G. Gassert

**Affiliations:** 1 Department of Diagnostic and Interventional Radiology, Klinikum rechts der Isar, Technische Universität München, Ismaninger Str. 22, 81675 Munich, Germany; 2 Department of Diagnostic and Interventional Neuroradiology, Klinikum rechts der Isar, Technische Universität München, Ismaninger Str. 22, 81675 Munich, Germany; 3 Department of Oral and Maxillofacial Surgery, University of Illinois at Chicago, Chicago, IL USA; 4 Department of Nuclear Medicine, Inselspital, University of Bern, Bern, Switzerland; 5 Department of Oral and Maxillofacial Surgery and Facial Plastic Surgery, Klinikum rechts der Isar, Technische Universität München, Munich, Germany; 6 Department of Radiology and Nuclear Medicine, University Medical Centre Mannheim, Theodor-Kutzer-Ufer 1-3, 68167 Mannheim, Germany; 7 Department of Radiology, University Hospital, LMU Munich, Marchioninistr. 15, 81377 Munich, Germany

**Keywords:** Cost effectiveness analysis, Head and neck cancer, Oncology, MRI, PET/CT, CT

## Abstract

**Background and purpose:**

Treatment of oral squamous cell carcinoma (OSCC) is based on clinical exam, biopsy, and a precise imaging-based TNM-evaluation. A high sensitivity and specificity for magnetic resonance imaging (MRI) and F-18 FDG PET/CT are reported for N-staging. Nevertheless, staging of oral squamous cell carcinoma is most often based on computed tomography (CT) scans. This study aims to evaluate cost-effectiveness of MRI and PET/CT compared to standard of care imaging in initial staging of OSCC within the US Healthcare System.

**Methods:**

A decision model was constructed using quality-adjusted life years (QALYs) and overall costs of different imaging strategies including a CT of the head, neck, and the thorax, MRI of the neck with CT of the thorax, and whole body F-18 FDG PET/CT using Markov transition simulations for different disease states. Input parameters were derived from literature and willingness to pay (WTP) was set to US $100,000/QALY. Deterministic sensitivity analysis of diagnostic parameters and costs was performed. Monte Carlo modeling was used for probabilistic sensitivity analysis.

**Results:**

In the base-case scenario, total costs were at US $239,628 for CT, US $240,001 for MRI, and US $239,131 for F-18 FDG PET/CT whereas the model yielded an effectiveness of 5.29 QALYs for CT, 5.30 QALYs for MRI, and 5.32 QALYs for F-18 FDG PET/CT respectively. F-18 FDG PET/CT was the most cost-effective strategy over MRI as well as CT, and MRI was the cost-effective strategy over CT. Deterministic and probabilistic sensitivity analysis showed high robustness of the model with incremental cost effectiveness ratio remaining below US $100,000/QALY for a wide range of variability of input parameters.

**Conclusion:**

F-18 FDG PET/CT is the most cost-effective strategy in the initial N-staging of OSCC when compared to MRI and CT. Despite less routine use, both whole body PET/CT and MRI are cost-effective modalities in the N-staging of OSCC. Based on these findings, the implementation of PET/CT for initial staging could be suggested to help reduce costs while increasing effectiveness in OSCC.

## Introduction

Oral squamous cell carcinoma (OSCC) is the most frequent cancer entity in the head and the neck region and among the most common oncologic diseases [1, 2]. Besides a thoroughly performed clinical exam, imaging plays a central role in diagnosing oral squamous carcinoma [3, 4]. These examinations consist of anamnesis (e.g., with regard to smoking habits) followed by intraoral inspection, neck palpation, and biopsy if necessary. Adequate diagnosis of local tumor extent and early detection of metastases are crucial for early disease detection, therapy monitoring, and clinical staging. Treatment of OSCC, including resection, radiation, and chemotherapy, is based on clinical exam, biopsy, and a precise imaging-based TNM-evaluation [5]. The current diagnostic standard involves computed tomography (CT) scan of the head and neck, and the chest. Recent studies however have reported the advantage of diffusion-based magnetic resonance imaging (MRI) sequences in detecting local disease extent, as well as F-18 fluorodeoxyglucose (FDG) positron emission tomography combined with CT (PET/CT) scanning in determining the extent of nodal spread [6, 7]. Previous studies have shown the high sensitivity and specificity of PET/CT in determining N-stage can avoid the need for additional diagnostic procedures [8].

Given the diagnostic advantages of F-18 FDG PET/CT with regard to detection of lymph node involvement and the precision of new MRI techniques for assessing local invasion, their cost-effectiveness has been assessed for treatment response evaluation but not for initial staging purposes [6]. With regard to determining preoperative surgical resection margins, inclusion of all available imaging is essential. Elective neck dissection (END) is often performed in patients with clinically unsuspicious cervical lymph node status, whereas modified radical neck dissection (MRND) is conducted when lymph node invasion is apparent. In most current guidelines, CT is the imaging modality of choice for initial staging of OSCC, including the USA [5]. In the USA, performing a PET/CT is associated with cost of US $1564.00, compared to US $956.00 for MRI (including neck MRI, CT of the thorax, and abdomen) and US $744.00 for CT (including neck, thorax, and abdomen) (source: The Surveillance, Epidemiology, and End Results (SEER) Program).

Implementing cost-effective imaging modalities is a high priority for patients, care-takers, and healthcare systems with growing medical and socioeconomic costs [9, 10]. The use of Markov models for the medical and economic advantages of various imaging modalities has previously been reported in the oncologic setting [11–14]. Despite literature showing higher accuracy of F-18 FDG PET/CT and MRI for detection of lymph node metastases as compared to standard of care imaging, so far, no study has investigated the cost-effectiveness of these modalities in initial N-staging of OSCC. Therefore, this study aims to evaluate cost-effectiveness of MRI and F-18 FDG PET/CT compared to standard of care imaging in initial staging of oral squamous cell carcinoma in the US Healthcare System.

## Materials and methods

### Model

**Fig. 1:**
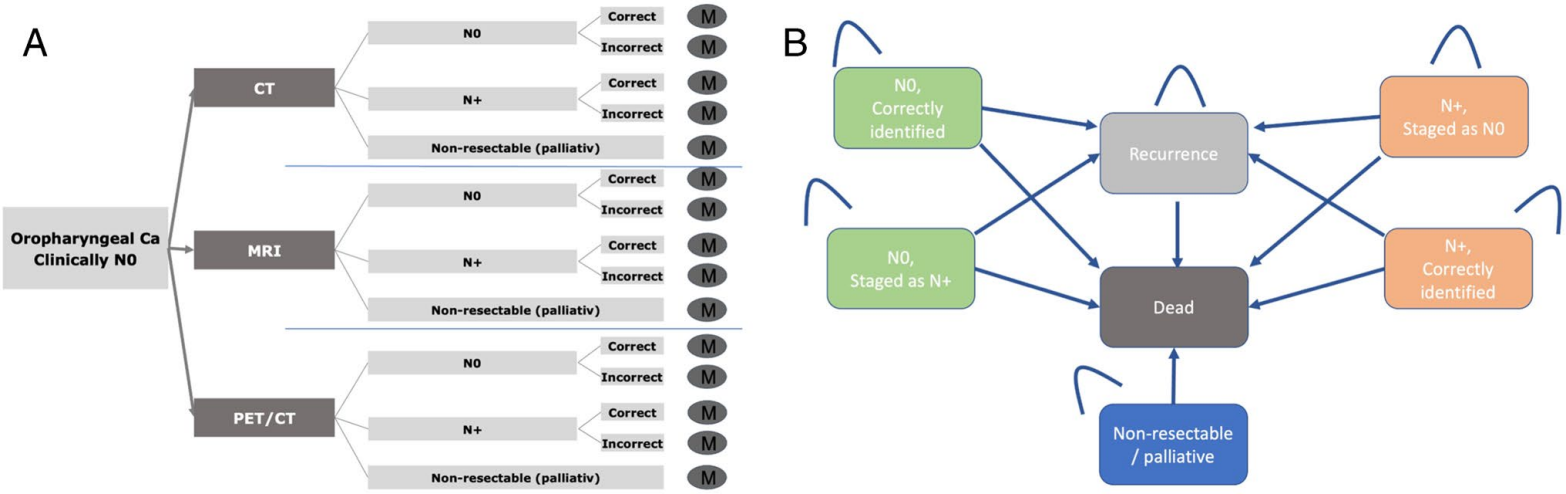
Overview of the decision model for the diagnostic strategies “CT,” ”MRI,” and “PET/CT” (**A**). For each diagnostic outcome, a Markov model analysis was performed (**B**). Different states and potential transitions are shown in the Markov model. The initial state was determined by the outcome in the decision model. TCT, computed tomography; MRI, magnetic resonance imaging; PET, positron emission tomography; N0, no lymphnode metastasis; N+, lymphnode metastasis

An economic decision tree was created including three diagnostic modalities: First, CT of the neck, chest, and abdomen, referred to as “CT.” Second, MRI of the neck + CT of chest and abdomen, referred to as “MRI.” Third, whole body F-18 FDG PET/CT, referred to as “PET/CT.” A Markov transition state model was created based on the possible outcomes after the diagnostic procedure and the respective sensitivities and specificities for the detection of lymph node metastases. The following states were applied as shown in Fig. 1:

- N0, identified as N0 (True negative)
- N0, identified as N+ (False positive)
- N+, identified as N+ (True positive)
- N+, identified as N0 (False negative)
- Non-resectable/palliative
- Recurrence
- Dead

Patients with M1 cancer were classified as non-resectable. For further analysis, only patients with M0 status were included.

### Input parameters

Review of literature was performed to identify recent (not older than 10 years) and reliable data for definition of model input parameters as shown in Table 1. Sensitivity and specificity values for evaluation of lymph-node status for the three modalities was adapted as described previously [15–17]. We identified low T and N stages in initial OSCC staging as a clinically relevant scenario, in which patients with morphologically normal appearing lymph nodes could benefit from functional imaging with F18 FDG-PET/CT. In recent literature, there is a paucity of data on pathologic lymph node morphology and cutoff values discerning reactive from neoplastic changes. Regarding identification of malignant cervical lymph nodes, functional imaging with F18 FDG-PET/CT was reported to be superior in sensitivity compared to MRI or CT alone [16]. Despite higher cost of F18 FDG-PET/CT, the long-term effects on healthcare systems of a possible implementation of this imaging modality for initial staging in OSCC are not clear. In our model, the acute and long-term costs of each imaging modality and their respective diagnostic performance where evaluated in a scenario of low T and N stages.

**Table 1:** Model input parameters

Variable	Estimate	Source
Pre-test probability of initial lymph node metastasis	62%	National Comprehensive Cancer Network [5]
Pre-test probability of initial distant metastasis	19%	National Comprehensive Cancer Network [5]
Expected age at diagnostic procedure	63 years	National Comprehensive Cancer Network [5]
Assumed willingness-to-pay per QALY	$100,000.00	Assumption
Discount rate	3%	Assumption
Markov model time horizon	10 years	Assumption
Diagnostic test performances		
Sensitivity for lymph node metastasis CT	81%	Nguyen et al. 2014 [16]
Specificity for lymph node metastasis CT	88%	Nguyen et al. 2014 [16]
Sensitivity for lymph node metastasis MRI	78%	Schaarschmidt et al. 2016 [15]
Specificity for lymph node metastasis MRI	99%	Schaarschmidt et al. 2016 [15]
Sensitivity for lymph node metastasis PET/CT	95%	Nguyen et al. 2014 [16]
Specificity for lymph node metastasis PET/CT	90%	Nguyen et al. 2014 [16]
Costs (Acute)		
CT (including CT neck/thorax/abdomen)	$744.00	Medicare (Ref.No.: 70491 + 71260 + 74160)
MRI (including MRI neck + CT thorax/abdomen)	$956.00	Medicare (Ref.No.: 70542 + 71260 + 74160)
PET/CT	$1,564.00	Medicare (Ref.No.: 78815)
Elective neck dissection	$17,291.00	Govers et al. 2015 [37]
Modified radical neck dissection	$18,642.00	Govers et al. 2015 [37]
Primary oral tumor resection + neck dissection + adjuvant radiochemo	$80,887.00	Acevedo et al. 2016 [38]
Costs (long-term)		
Follow-up post resection	$1,362.00	Acevedo et al. 2016 [38]
Non-resectable/palliative	$59,438.00	Lafuma et al. 2019 [39]
Recurrence	$59,438.00	Lafuma et al. 2019 [39]
Utilities		
Post-resection, tumor-free after elective neck dissection	0.913	Acevedo et al. 2016 [38]
Post-resection, tumor-free after modified radical	−0.072	Acevedo et al. 2016 [38]
Recurrence	−0.343	Acevedo et al. 2016 [38]
Loss in QoL due to surgery	−0.06	Govers et al. 2015 [37]
Death	0	Assumption
Transition probabilities		
Recurrence after correctly identified N0	12.8%	Feng et al. 2014 [40]
Recurrence after wrongly modified radical neck dissection in N0	12.8%	Feng et al. 2014 [40]
Recurrence after correct modified radical neck dissection in N+	12.8%	Feng et al. 2014 [40]
Recurrence after only elective neck dissection in N+	15.7%	Feng et al. 2014 [40]

The age-dependent risk of death was adopted from the US Life Tables endorsed by the Centers for Disease Control and Prevention, National Center for Health Statistics, and National Vital Statistics System [18]. Similar risk of death by other causes was assumed between patients in all groups. Probability of recurrence after neck dissection was set to 15.7% for END and 12.8% for MRND as described previously [19]. Risk of death with metastasized diseased (initially and after recurrence) was derived from literature and set to 17%/year (Source: SEER Medicare Dart).

This study was based on the US Healthcare System. Therefore, costs of diagnostic modalities were derived from Medicare and all costs are indicated in US-$. Respective reference numbers are shown in Table 1. Costs for “Elective neck dissection” and “Modified radical neck dissection” as well as long-term costs were based on review of recent literature. Long-term costs of tumor-free patients derive from follow-up examinations.

Quality of life (QoL) was determined for each state of the Markov model. Utility was then measured in quality-adjusted life years (QALY) through diagnostic procedures. QoL was set to 0.913 for patients after tumor resection with END based on previous literature. QoL was reduced by 0.072 for patients who received MRND due to larger resection and by 0.343 for patients with recurrent disease as described previously. Additionally, QoL was assumed to be reduced by 0.1 in the first year after surgery due to surgical procedure.

### Economic analysis

A dedicated decision analysis software (TreeAge Pro Version 19.1.1, Williamstown, MA, USA) was used for further analysis and a discount rate of 3.0% was assumed according to current recommendations [20]. Willingness to pay (WTP) was set to US $100,000 per QALY. Based on current guidelines and the 5-year survival rates of OSCC, the total time horizon of the analysis was set to 10 years after initial diagnosis [20]. Cycle length was set to 1 year resulting in 10 iterations for model-simulation.

To study the impact of possible variation of input parameters on the incremental cost-effectiveness ratio (ICER), a deterministic sensitivity analysis was performed.

The incremental cost-effectiveness ratio reflects the required WTP so that an alternative method stays cost-effective. Range for variation was chosen based on previous literature with diagnostic test performances variation of at least 0.05 and cost of the three diagnostic modalities analyzed varying by at least 15%. Variation of probability of initial lymph node metastasis was based on expert opinion and therefore chosen in a wide range. Additionally, influence of variation of probability of recurrence was examined.

Input parameters are indicated as a firm value each. Nevertheless, these input parameters vary among individuals although the correct mean is indicated (e.g., “Expected age at diagnostic procedure” is indicated as 63 years, although also older and younger patients are included in this analysis). For analysis of this overall uncertainty of the input parameters and their combined impact on cost-effectiveness, a probabilistic sensitivity analysis was performed based on the probability distributions (the γ-distribution was used for the cost-parameters, the β-distribution for all other parameters). Monte-Carlo modeling with a total of 30,000 iterations was used for calculation of the model.

## Results

### Cost-effectiveness analysis

As studies showed high accuracy of F-18 FDG PET/CT and MRI for detection of lymph node metastases as compared to standard of care imaging in OSCC, the goal of this study was to evaluate cost-effectiveness of these diagnostic modalities in initial staging of OSCC in the US Healthcare System.

Applying a WTP of US $100,000 per QALY and a time horizon of 10 years, the strategy “CT” resulted in total costs of US $239,628 and an expected effectiveness of 5.29 QALYs. The strategy “MRI” resulted in total costs of US $240,001 and an expected effectiveness of also 5.30 QALYs whereas total costs and expected effectiveness were at US $239,131 and 5.32 QALYs for the strategy “PET/CT” respectively. Therefore, from an economic point of view, the strategies “CT” and “MRI” were dominated by “PET/CT.” In the base case scenario, ICER was negative and did not cross the WTP of US $100,000 per QALY for both cases, “PET/CT” compared to “CT” and at MRI compared to “CT” reflecting both lower overall costs and higher effectiveness when applying “PET/CT.”

### Deterministic sensitivity analysis

**Fig. 2:**
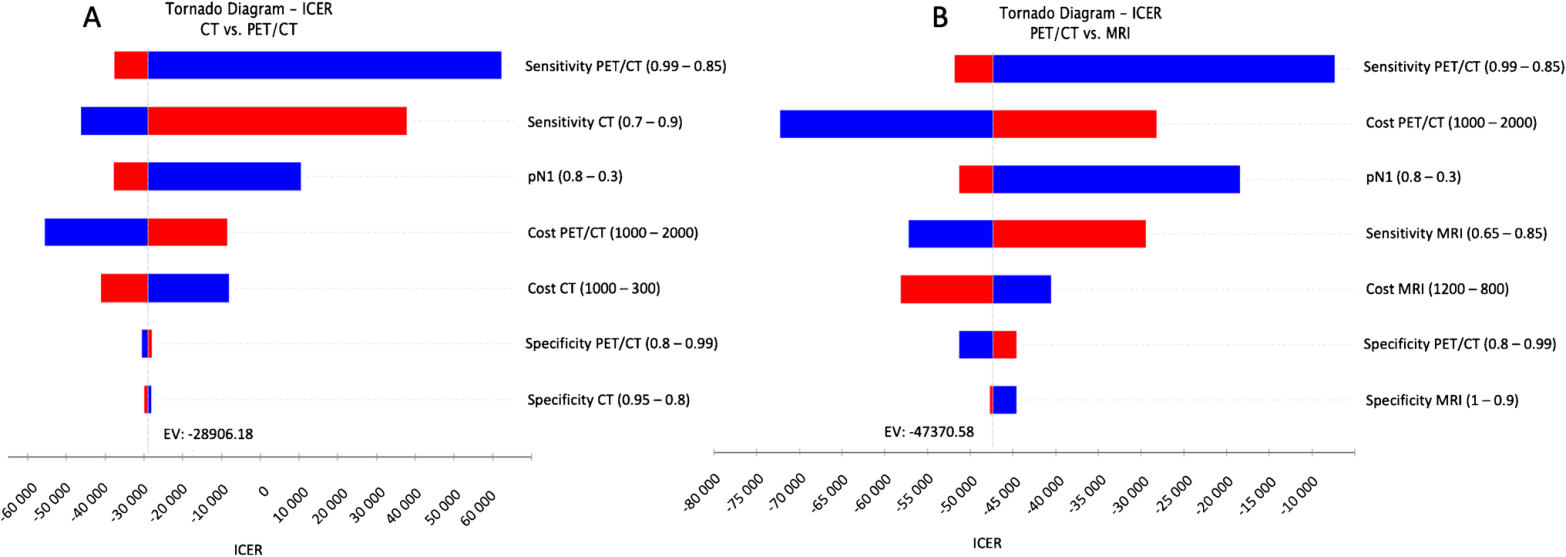
Results of the deterministic sensitivity analysis visualized as a tornado diagram. Bars indicate the impact of variation of input parameters on incremental cost-effectiveness ratio (ICER) starting from expected value in base case scenario for the comparison of PET/CT vs. CT (**A**) and PET/CT vs MRI (**B**). For all parameters investigated the ICER remains below the WTP of $100,000/QALY in both comparisons. CT, computed tomography; MRI, magnetic resonance imaging; PET, positron emission tomography, M1, with metastases, N1, lymphnode metastases

Input parameters were derived from a recent literature. Nevertheless, as input parameters are derived from literature, possible errors and variation of input parameters and influence on the incremental cost-effectiveness ratio should be considered. To account for variation of diagnostic accuracies as well as costs of diagnostic modalities, a deterministic sensitivity analysis is performed as shown in Fig. 2.

Applying wide ranges of 0.65 to 0.85/0.7 to 0.9 for sensitivity and 0.9 to 1/0.7 to 0.9 for specificity of “MRI” and “CT” and of 0.85 to 0.99 for sensitivity and 0.8 to 0.99 for specificity of “PET/CT,” “PET/CT” stayed the dominant strategy as ICER remained below the WTP of US $100,000 per QALY. Additionally, influence of variation of probability of initial presence of lymph node metastasis was analyzed, showing robustness of the model to differing probability of initial lymph node metastasis between 30 and 80%.

Variation of values of probability of recurrence showed relatively high influence on the economic model. When performing a cutoff analysis, PET/CT lost its dominance over CT at a probability of recurrence of 0.134 and 0.151 after END and MRND, respectively. As compared to MRI, PET/CT lost its dominance at a probability of recurrence of 0.136 and 0.148 after END and MRND, respectively.

### Probabilistic sensitivity analysis

**Fig. 3:**
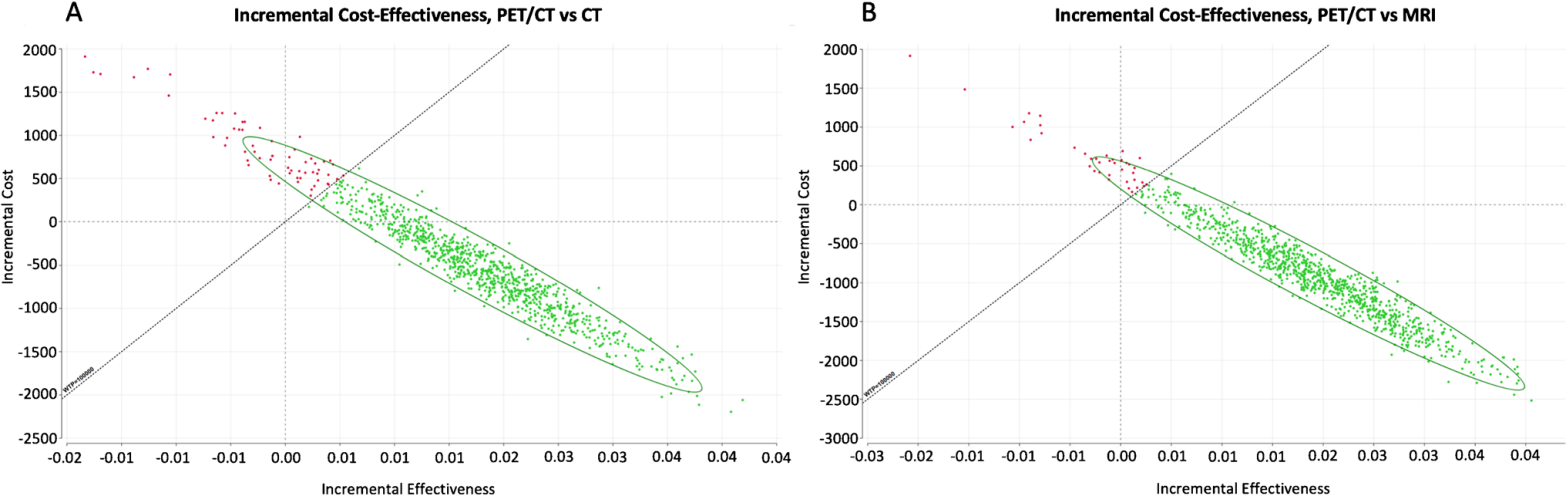
Scatterplot of Monte-Carlo simulation showing incremental cost and incremental effectiveness of PET/CT compared to CT (**A**) and PET/CT compared to MRI (**B**) for exemplary iterations. CT, computed tomography; MRI, magnetic resonance imaging; PET, positron emission tomography; WTP, willingness to pay

To account for model uncertainties and validate the model, probabilistic sensitivity analysis using Monte-Carlo simulations was performed including all three modalities. Comparison of distributions between PET/CT and CT as well as PET/CT and MRI is visualized in Fig. 3.

Within a range of US $0 to US $200,000 for the WTP, majority of iterations resulted in higher amount of iterations cost-effective for PET/CT than for MRI or CT as illustrated by a cost-effectiveness acceptability curve (Fig. 5). At a WTP of US $100,000, 88.8% of simulations were cost-effective for PET/CT, 7.5% for CT and 3.6% for MRI.

## Discussion

OSCC is the most frequent neoplastic entity in the head and neck region and requires the collaboration of several medical disciplines, including radiologists, oncologists, and surgeons. These specialists rely on various imaging modalities for the diagnosis and treatment in order to maintain form and function of the oropharynx [21]. Precise imaging for the initial assessment of head and neck cancer therefore plays a pivotal role in this respect. Recently, increased attention has been given to identification of cost-effective imaging modalities with high diagnostic validity [9, 10]. This study provides initial evidence to support the use of PET/CT and MRI as cost-effective diagnostic imaging modalities for initial staging of lymph node invasion in OSCC. While the technical superiority of MRI compared to CT in local extent determination and the excellent negative predictive value of PET/CT were shown before, the suitability of MRI for initial staging purposes has not been investigated under an economic point of view [22, 23].

In our study, the ICER is negative for PET/CT compared to CT as well as compared to MRI, showing lower overall costs and higher effectiveness. Using deterministic sensitivity analysis, we show that the ICER remains below the WTP of US $100,000/QALY for different variations in the input parameters, showing robustness of our model and underlining cost-effectiveness in a wide range of individual patient scenarios.

Our results concur with current literature showing cost-effectiveness of the initially more cost-intensive PET-imaging in different oncologic diseases [14, 24, 25]. Previous studies showed PET/CT can be a cost-effective modality to assess treatment response to adjuvant radiation, and that PET/CT can be utilized during follow-up as a cost-effective modality to assess recurrence, thereby avoiding unnecessary surgical harm [8, 23, 26]. While diagnostic accuracy of MRI has proven superior in visualizing local tissue infiltration, its cost-effectiveness has currently been restricted to advanced T3 to T4 cancer stages [4, 27]. However, our study has shown the utility of MRI in detecting morphologically benign, deep lymph node metastasis, which is critical to surgical management and the reduction of recurrence rates.

Prior to surgery, biopsy and imaging are reviewed for depth of invasion and lymphatic spread, guiding surgical management with END versus MRND. In contrast to patients with N0 disease, who have been shown to benefit from less radical END, the extent of neck dissection needed for patients with OSCC with positive cervical lymphadenopathy remains controversial [28]. There are studies which support performing END for N2 OSCC and display comparably low recurrence rates, but patient age, risk factors, comorbidities, and disease characteristics must be considered [29, 30]. In contrast, some studies show a group of patients with N2 OSCC will have a better outcome if treated with MRND, taking into consideration disease characteristics such as perineural or lymphovascular invasion [28].

Regarding local tumor extent, there are considerations that have to be taken into account before therapy regimen planning. When advanced infiltration including osseous and nervous structures is present overall survival is reduced as lower T stages (T1) are associated with better prognosis [31]. Additionally to T staging, a recent study conducted by Suresh et al. showed that the site of OSCC also plays a pivotal role with regard to disease free survival and overall survival. In this study, tumors of the tongue displayed a better prognosis than tumors of the retromolar trigone [31]. However, it has to be stated that specific invasion patterns cannot be considered in interdisciplinary questions focusing on the interface of economics and imaging as the main goal of such approaches is to cover the average clinical constellations.

Besides initial imaging in therapy naive patients, incorporating PET-CT before adjuvant irradiation in OSCC with suspected advanced lymph node metastasis could result in lower recurrence and false negative lymph node status [32–34]. Zhou et al. also displayed how annual MRI in patients with nasopharyngeal carcinoma and lymphatic metastasis after irradiation is a cost-effective strategy for T3-4 patients, depending on the social willingness to pay [27]. However, in early post-treatment imaging, using both MRI as well as PET/CT for differentiation of local recurrence from postoperative fibrotic tissue alterations or edematous changes is feasible but can be demanding [27, 35].

There are several methodological limitations in our study. The study adhered to the recommended guidelines for conceptualizing and performing cost-effectiveness analysis; however, these are based on modeling iterations and input parameters [20, 36]. The decision-based model is prone to deviating results when input parameters change, especially on recurrence rates after END and MRND as our sensitivity analysis displayed. Second, local OSCC extent and local invasion depth were not included in our analysis as this would require additional numerations beyond the scope of this study. Third, in the model-based approach, no individual patient history or further accompanying diseases are taken into account, and cost-effectiveness analyses were performed for an average of patients, without any individual considerations. Therefore, results reflect populations and not the individual and can be applied by healthcare entities but would not substitute an individual’s decision-making. Healthcare organizations and insurance companies could utilize this information to further increase access to MRI and PET/CT. On the other hand, an individual should consider their personal access to MRI or PET/CT, such as travel, out of pocket costs, and insurance coverage. Additionally — a limitation applying to any cost-effectiveness analysis — our study focuses on the US Healthcare System and due to different costs of procedures and therapies results are not easily transferable to other healthcare systems. Our study included CT, MRI, and PET/CT as these modalities are available in most health care institutions. Future cost-effectiveness studies could include advanced imaging techniques like PET/MRI combining the advantages of both imaging modalities. Nevertheless, previous studies for reliable determination of accuracy PET/MRI are necessary.

Future studies on cost-effectiveness could analyze alternative imaging techniques, such as PET/MRI, which high costs may be offset by high diagnostic accuracy. In order to perform this analysis of PET/MRI, however, reliable studies on the accuracy of PET/MRI must first be completed. Given the existing literature, we can only make an assessment at this time of PET-CT and MRI.

## Conclusion

This study evaluates the cost-effectiveness of MRI and F-18 FDG PET/CT compared to standard of care imaging in the initial staging of oral squamous cell carcinoma in the USA. The results display F-18 FDG PET/CT and MRI as cost-effective imaging strategies for initial N-staging in oral squamous cell carcinoma with high robustness to variation of input parameters. The findings have medical and economic impact on the diagnostic work-up in imaging of OSCC and suggest the implementation of PET/CT as the standard of care imaging for initial staging of oral squamous cell carcinoma. With ongoing technological advancements of PET/CT and MRI, such as increased sensitivity and specificity with improved resolution, future analysis may further support these modalities and help reduce healthcare costs while increasing effectiveness in patients with oral squamous cell carcinoma.

## References

[1] Miranda-Filho A, Bray F (2020). Global patterns and trends in cancers of the lip, tongue and mouth. Oral Oncol.

[2] Megwalu UC, Sirjani D, Devine EE (2018). Oropharyngeal squamous cell carcinoma incidence and mortality trends in the United States, 1973–2013. Laryngoscope.

[3] Warnakulasuriya S, Kerr AR (2021). Oral cancer screening: past, present, and future. J Dent Res.

[4] Vaid S, Chandorkar A, Atre A, Shah D (2017). Differentiating recurrent tumours from post-treatment changes in head and neck cancers: does diffusion-weighted MRI solve the eternal dilemma?. Clin Radiol.

[5] Caudell JJ, Gillison ML, Maghami E, Spencer S, et al. NCCN guidelines(R) insights: head and neck cancers, Version 1.2022. J Natl Compr Canc Netw. 2022;20(3):224–234. 10.6004/jnccn.2022.0016 35276673

[6] Greuter MJ, Schouten CS, Castelijns JA, de Graaf P (2017). Cost-effectiveness of response evaluation after chemoradiation in patients with advanced oropharyngeal cancer using (18)F-FDG-PET-CT and/or diffusion-weighted MRI. BMC Cancer.

[7] Wiener E, Pautke C, Link TM, Neff A (2006). Comparison of 16-slice MSCT and MRI in the assessment of squamous cell carcinoma of the oral cavity. Eur J Radiol.

[8] Mehanna H, Wong WL, McConkey CC, Rahman JK (2016). PET-CT surveillance versus neck dissection in advanced head and neck cancer. N Engl J Med.

[9] Wissinger E, Griebsch I, Lungershausen J, Foster T (2014). The economic burden of head and neck cancer: a systematic literature review. Pharmacoeconomics.

[10] Brouwer W, van Baal P, van Exel J, Versteegh M (2019). When is it too expensive? Cost-effectiveness thresholds and health care decision-making. Eur J Health Econ.

[11] Froelich MF, Schnitzer ML, Rathmann N, Tollens F, et al. Cost-effectiveness analysis of local ablation and surgery for liver metastases of oligometastatic colorectal cancer. Cancers (Basel). 2021;13(7). 10.3390/cancers13071507 PMC8037107 33806059

[12] Froelich MF, Schnitzer ML, Holzgreve A, Gassert FG, et al. Cost-effectiveness analysis of (68)Ga DOTA-TATE PET/CT, (111)In-Pentetreotide SPECT/CT and CT for Diagnostic Workup of Neuroendocrine Tumors. Diagnostics (Basel). 2021;11(2). 10.3390/diagnostics11020334 PMC7922846 33670457

[13] Froelich MF, Kunz WG, Tollens F, Schnitzer ML, et al. Cost-effectiveness analysis in radiology: methods, results and implications. Rofo. 2021;194(1):29–38. 10.1055/a-1502-7830 34139781

[14] Gassert FG, Rubenthaler J, Cyran CC, Rink JS (2021). (18)F FDG PET/MRI with hepatocyte-specific contrast agent for M staging of rectal cancer: a primary economic evaluation. Eur J Nucl Med Mol Imaging.

[15] Schaarschmidt BM, Heusch P, Buchbender C, Ruhlmann M (2016). Locoregional tumour evaluation of squamous cell carcinoma in the head and neck area: a comparison between MRI, PET/CT and integrated PET/MRI. Eur J Nucl Med Mol Imaging.

[16] Nguyen A, Luginbuhl A, Cognetti D, Van Abel K (2014). Effectiveness of PET/CT in the preoperative evaluation of neck disease. Laryngoscope.

[17] Kim JH, Choi KY, Lee SH, Lee DJ (2020). The value of CT, MRI, and PET-CT in detecting retropharyngeal lymph node metastasis of head and neck squamous cell carcinoma. BMC Med Imaging.

[18] Arias E, Xu J, Kochanek KD (2019). United States life tables, 2016. Natl Vital Stat Rep.

[19] Feng Z, Niu LX, Yuan Y, Peng X (2014). Risk factors and treatment of contralateral neck recurrence for unilateral oral squamous cell carcinoma: a retrospective study of 1482 cases. Oral Oncol.

[20] Sanders GD, Neumann PJ, Basu A, Brock DW (2016). Recommendations for conduct, methodological practices, and reporting of cost-effectiveness analyses: second panel on cost-effectiveness in health and medicine. JAMA.

[21] Argiris A, Karamouzis MV, Raben D, Ferris RL (2008). Head and neck cancer. Lancet.

[22] Schouten CS, de Graaf P, Alberts FM, Hoekstra OS (2015). Response evaluation after chemoradiotherapy for advanced nodal disease in head and neck cancer using diffusion-weighted MRI and 18F-FDG-PET-CT. Oral Oncol.

[23] Marcus C, Ciarallo A, Tahari AK, Mena E (2014). Head and neck PET/CT: therapy response interpretation criteria (Hopkins Criteria)-interreader reliability, accuracy, and survival outcomes. J Nucl Med.

[24] Dieng M, Khanna N, Nguyen MTH, Turner R (2020). Cost-effectiveness analysis of PET/CT surveillance imaging to detect systemic recurrence in resected stage III melanoma: study protocol. BMJ Open.

[25] Schreyogg J, Weller J, Stargardt T, Herrmann K (2010). Cost-effectiveness of hybrid PET/CT for staging of non-small cell lung cancer. J Nucl Med.

[26] Sher DJ, Tishler RB, Annino D, Punglia RS (2010). Cost-effectiveness of CT and PET-CT for determining the need for adjuvant neck dissection in locally advanced head and neck cancer. Ann Oncol.

[27] Zhou GQ, Wu CF, Zhang J, Mao YP (2018). Cost-effectiveness analysis of routine magnetic resonance imaging in the follow-up of patients with nasopharyngeal carcinoma after intensity modulated radiation therapy. Int J Radiat Oncol Biol Phys.

[28] Schiff BA, Roberts DB, El-Naggar A, Garden AS (2005). Selective vs modified radical neck dissection and postoperative radiotherapy vs observation in the treatment of squamous cell carcinoma of the oral tongue. Arch Otolaryngol Head Neck Surg.

[29] Kowalski LP, Carvalho AL (2002). Feasibility of supraomohyoid neck dissection in N1 and N2a oral cancer patients. Head Neck.

[30] Traynor SJ, Cohen JI, Gray J, Andersen PE (1996). Selective neck dissection and the management of the node-positive neck. Am J Surg.

[31] Suresh GM, Koppad R, Prakash BV, Sabitha KS (2019). Prognostic indicators of oral squamous cell carcinoma. Ann Maxillofac Surg.

[32] Roh JL, Park JP, Kim JS, Lee JH (2014). 18F fluorodeoxyglucose PET/CT in head and neck squamous cell carcinoma with negative neck palpation findings: a prospective study. Radiology.

[33] Liao CT, Fan KH, Lin CY, Wang HM (2012). Impact of a second FDG PET scan before adjuvant therapy for the early detection of residual/relapsing tumours in high-risk patients with oral cavity cancer and pathological extracapsular spread. Eur J Nucl Med Mol Imaging.

[34] Jwa E, Lee SW, Kim JS, Park JH (2012). Prognostic value of (18)F-fluorodeoxyglucose positron emission tomography, computed tomography and magnetic resonance imaging in oral cavity squamous cell carcinoma with pathologically positive neck lymph node. Radiat Oncol J.

[35] Chong VF, Ong CK (2008). Nasopharyngeal carcinoma. Eur J Radiol.

[36] Sonnenberg FA, Beck JR (1993). Markov models in medical decision making: a practical guide. Med Decis Making.

[37] Govers TM, Patel S, Takes RP, Merkx T (2015). Cost-effectiveness of selective neck dissection versus modified radical neck dissection for treating metastases in patients with oral cavity cancer: A modelling study. Head Neck.

[38] Acevedo JR, Fero KE, Wilson B, Sacco AG (2016). Cost-effectiveness analysis of elective neck dissection in patients with clinically node-negative oral cavity cancer. J Clin Oncol.

[39] Lafuma A, Cotte FE, Le Tourneau C, Emery C (2019). Economic burden of chemotherapy-treated recurrent and/or metastatic squamous cell carcinoma of the head and neck in France: real-world data from the permanent sample of national health insurance beneficiaries. J Med Econ.

[40] Feng Z, Li JN, Niu LX, Guo CB (2014). Supraomohyoid neck dissection in the management of oral squamous cell carcinoma: special consideration for skip metastases at level IV or V. J Oral Maxillofac Surg.

